# Faecal calprotectin: factors affecting levels and its potential role as a surrogate marker for risk of development of Crohn’s Disease

**DOI:** 10.1186/s12876-016-0535-z

**Published:** 2016-10-07

**Authors:** Michael A. Mendall, Derek Chan, Roshani Patel, Devinder Kumar

**Affiliations:** 1 Department of Gastroenterology, Croydon University Hospital, 530 London Road, Surrey, CR7 7YE UK; 2 Department of Gastroenterology, Blackshaw Road, London, SW17 0QT UK; 3 Department of Colorectal Surgery, St George’s Hospital, Blackshaw Road, London, SW17 0QT UK

**Keywords:** Crohn’s disease, Calprotectin, Surrogate marker, Environment, Diet

## Abstract

**Background:**

Faecal calprotectin (FC) is one of the most widely used non-invasive tests for the diagnosis and assessment of Crohn’s disease (CD) activity. Despite this, factors other than disease activity which affect levels have not been extensively reviewed. This is of importance when using FC in the diagnostic setting but also may be of utility in studying the aetiology of disease.

**Objectives:**

Our review outlines environmental risk factors that affect FC levels influencing diagnostic accuracy and how these may be associated with risk of developing CD. FC as a surrogate marker could be used to validate risk factors established in case control studies where prospective studies are not feasible. Proof of this concept is provided by our identification of obesity as being associated with elevated FC, our subsequent confirmation of obesity as risk factor for CD and the subsequent verification in prospective studies, as well as associations of lack of physical activity and dietary fibre intake with elevated FC levels and their subsequent confirmation as risk factors in prospective studies.

**Conclusion:**

We believe that FC is likely to prove a useful surrogate marker for risk of developing CD. This review has given a theoretical basis for considering the epidemiological determinants of CD which to date has been missing.

## Background

Faecal calprotectin (FC) is one of the most widely used non-invasive tests for the diagnosis and assessment of Crohn’s disease (CD) activity. Despite this, factors other than the presence of disease and disease activity that affect levels, have not been adequately reviewed. This is important particularly in the context of the use of FC in assessing the risk that a patient with symptoms may have disease.

Another potentially important issue is the question as to whether FC levels may be of use in establishing a subject’s risk of developing Crohn’s disease in the future. This possibility will also be further elaborated on in this review, as factors influencing levels in asymptomatic subjects may also have a bearing on this.

CD is a complex chronic disease. Over the last decade large population studies and experimental studies have helped to elucidate the aetiopathogenesis of the disease. The onset of CD is caused by an interaction of genetic susceptibility, environmental triggers and subsequent change in the intestinal microbiome causing disruption of the host immunity [1]. As with many other diseases, the relative contribution of environment over genetic influences increases with age. With early onset CD, genetics are likely to play an important role, whereas in later onset disease the effect of the environment is likely to be more prominent.

Studies of environment as a risk factor for disease are more difficult than genetic studies, particularly for relatively uncommon disorders such as CD where case control studies are the principle tools of investigation. Case control studies are prone to many types of bias, such as: recall bias, missing data, selection bias and temporal bias [2].

More reliable prospective cohort studies have a number of weaknesses: they require large numbers of subjects to be followed for long periods of time entailing a high economic cost. The induction period between risk factors and onset of disease is not yet known, making prospective follow up lengthy. Detailed information about exposure is not possible to get retrospectively, and importantly, prospective studies across the whole age range of CD presentation are not readily available.

What is needed is a surrogate marker for risk of developing CD. Surrogate markers have been widely used in the more prevalent cardiovascular diseases such as blood lipids and inflammatory markers. They allow detailed study of environmental exposures and more rapid hypothesis generation, avoiding the pit falls or complementing case control studies and compensating for the limitations of prospective cohort studies. Importantly they allow the study of interventions designed to mitigate risk. A good example in CD would be with the effect of detailed dietary components as commented upon recently by Kaplan GG, where it would be useful to be able to study the role of different types of fibre on risk of developing CD [3]. Additionally, a surrogate marker could be used to validate risk factors established in case control studies where prospective studies are not feasible.

FC maybe a useful surrogate marker of developing CD as suggested by family studies. These have shown increased lifetime risk of developing CD in children whose parents have CD than the general population [4]. Additionally non-affected family members who are known to be at increased risk, have consistently shown to have increased levels of FC; 49 % of relatives but only 13 % of spouses (who did not actually have CD) [5]. 5-10 % of first degree relatives will go on to develop CD [6]. Additionally, elevated FC is a well validated marker of risk of relapse in established CD in remission, which whilst is a different situation to risk of developing disease is likely to share some aetiological factors in common such as cigarette smoking [7, 8].

The remainder of this review explores the environmental factors which affect FC levels in subjects without disease and argues that as well as being an excellent marker of disease activity and relapse risk, may also be a candidate surrogate marker of risk of developing CD in subjects yet to develop the disease. We will make this argument by demonstrating that many environmental exposures associated with elevated FC in healthy subjects are themselves risk factors for CD. We will begin by assessing what is known about the determinants of FC levels in healthy subjects.

### Faecal calprotectin

Calprotectin is a 36 kDa Calcium and zinc binding protein expressed by neutrophils. FC concentration is raised due to neutrophil aggregation in the mucosa on inflamed intestine; it is therefore useful as a non-invasive marker for inflammation of inflammatory bowel disease (IBD). Levels correlate well with Indium white cell scanning and also gut permeability measured by other means [9]. There has been a great deal of interest in gut permeability in the aetiology of CD both in experimental and clinical studies and many of the findings with gut permeability are being replicated with FC [10–12]. The measurement of gut permeability itself however is cumbersome and not well suited to large-scale studies. Additionally, it is likely that gut permeability is merely a measure of gut mucosal inflammation, which is more related to the pathogenesis of CD. FC testing is non-invasive, stable, simple, easy to perform, rapid and reproducible. Importantly it is highly specific for intestinal inflammation. Other factors that affect levels will be reviewed shortly.

FC is now a well-established surrogate marker for diagnosing the onset of inflammatory bowel disease [13]. In the United Kingdom, National Institute of Clinical Excellence guidelines recommend its use to differentiate between functional gut disorders and a pathological organic cause of disease. It is also a useful in monitoring therapeutic management, prognosis and detecting relapse [14–17]. Although the sensitivity of FC in detecting intestinal inflammation is consistently high, in adults in developed world populations the specificity is only in the order of 80 % [13, 18]. Hence in a population with a relatively low prevalence of IBD diagnostic accuracy is relatively poor.

Levels are also elevated in other diseases such as gastroenteritis, gastrointestinal malignancies, reflux disease, cystic fibrosis and diverticulitis [13]. Despite this, little is known about the determinants of levels in normal subjects, and whether sensitivity and specificity vary with the age of the subject being tested.

### Environmental factors and faecal calprotectin

In a middle-aged healthy general population sample of 300 subjects (50-70 years old) our group has previously demonstrated that FC levels are independently elevated in association with environmental exposures such as low fibre intake, lack of physical exercise and increasing age [19]. The consumption of fibre and specifically vegetable fibre was found to have an inverse relationship with FC levels. There was a significant difference in the geometric mean FC (μg/g) between the lowest fibre quartile (which consumed < 9.8 % fibre versus the highest fibre quartile group (which consumed 17 to 38.8 % fibre). There was a 31 % increase in FC per decade increase in age, a 40 % increase per increase in body mass index (BMI) by 10 kg/m2, a 31 % decrease in those who exercised regularly, and a 2 % decrease for each percentage increase in proportion of the diet that was fibre consumption. All ofbody mass index these changes were significant after mutual adjustment. Trends were seen in cigarette consumption with a 7 % increase per pack year of smoking and a trend with vegetable intake with a 10 % decrease per daily portions consumed. There was no relation to proportion of the diet consumed as fruit or fat. There was a suggestion of U shaped relation with BMI. The lowest quartile (BMI 17.6 to 23.5) had a geometric mean FC level of 30 μg/g; the second quartile (BMI 23.6 to 25.6) mean FC of approximately 26 μg/g; the third quartile (BMI 25.7-28.1) had a mean FC of approximately 30 μg/g and the highest BMI quartile (BMI 28.2-41.2) had an FC of approximately 40 μg/g. Even without allowing for the likely u shaped relationship there was a significant positive association with BMI as a continuous variable. Also from the same cohort proton pump inhibitor (PPI) usage was found to be associated with elevated levels (geometric mean 78.16 ug/g vs. 30.9 ug/g *p* < 0.0001) [20].

For a test that is being widely used, it is surprising that so little work has been done on the determinants of normal levels. No other group has looked at the associations with exercise and fibre or other dietary components but a few small studies have assessed the association of FC with obesity with mixed results.

A small study of 13 obese adult subjects did not find confirmation of an association with FC, whereas a study 34 children with severe obesity did [21, 22]. Another small study again in adolescents showed only a small difference in FC between obese and normal subjects, but the obese subjects were significantly younger than the normal weight subjects and the children were not severely obese [23]. Finally a study in 28 adults with a range of weights demonstrated detectable FC only in obese subjects with an obese pattern of microbiota [24].

In a recent interventional study exploring the effect of weight loss in a community population, FC levels were found to fall with weight loss only in those with elevated levels at baseline [23]. Again, this was a small study.

These small experimental studies provide partial support to our observation in the only large study to date on the determinants of FC, though larger confirmatory studies are still required.

### Possible mechanisms for associations of FC with environmental factors

Low fibre intake is also associated with elevated circulating inflammatory markers [25]. The mechanism could be through alterations in bowel flora or nutrient density [26, 27]. The reduction with physical activity we postulated could be through the enhanced vagal tone that goes with physical fitness. The vagus nerve is known to have anti-inflammatory effects on the gut or reduced sympathetic tone that goes with increased vagal tone could again result in reduced inflammation [28]. There are also reports that exercise can alter bowel flora [29, 30].

Elevated BMI, which has been shown to be associated with increased gut permeability in both animal and human studies could be associated through a variety of mechanisms, including altered bowel flora, the effects of circulating inflammatory cells and markers or through direct effects of dietary fats on local cytokine production [30, 31]. Supporting the idea that altered bowel flora in the obese could mediate obesity associated elevations on FC, is a study in subjects with a variety of BMIs. This showed there was a distinct obesity related microbial profile and that it was this profile that was associated with elevated FC. Specifically in the obese microbiota profile there was reduced bacterial diversity, a decreased Bacteroides to Firmicutes ratio and an increased abundance of proinflammatory Proteobacteria [24].

### Environmental risk factors and Crohns disease

There are two distinct groups of risk factors for developing CD; those relating to the adult environment likely to be important in later onset CD and those relating to the early childhood environment likely to be of more relevance to early onset disease [32, 33]. There is little known about the determinants of FC in normal children and hence this review will not address risk factors in this age group further.

### Faecal Calprotectin as a surrogate marker for risk of developing Crohns disease

The evidence that FC may be a useful surrogate marker for risk of developing CD will now be reviewed by examining the evidence that exposures associated with changes in FC are similarly associated with altered risk of devloping CD. More recently powerful evidence from prospective cohort studies is emerging on the role of the environment in risk of developing CD, clarifying much of the earlier contradictory evidence about environment [34–40].

We will start by evaluating the evidence that abdominal obesity may be risk factor for developing CD. This hypothesis has been suggested by our original observation, that FC increases with increasing BMI in middle-aged healthy adults. This is a powerful example of the potential for utilizing FC as a marker of risk of developing CD as the association between CD and high BMI seems counterintuitive.

### Obesity

Obesity has been described as a global pandemic and is now a high priority action point in public health [41].

The first evidence that obesity may be a risk factor for developing CD came from our case control study of 524 patients [42]. This demonstrated a U shaped relationship between BMI at diagnosis and CD risk development; participants with CD were more likely to present with extremely low or extremely high BMI compared to subjects with ulcerative colitis (UC). Furthermore most of the obese subjects were in the older age group. There was a graded dose response of risk, with risk being highest in the most obese [38].

This evidence has been consolidated in a prospective study of the Danish National Birth Cohort of 75,008 women with 138 incident cases of CD, which found a U shaped dose-response relationship between BMI and risk of CD development, which was highest in those of low BMI and very high BMI [43]. There was median follow-up of 11 years and median age at start of follow-up was 30.2 years (27.2-33). Therefore it would be too premature to observe Montreal classification A3 disease (>40 yrs age at disease onset) in this cohort [44]. Further follow up studies would be interesting to examine CD in the older population or A3 group.

Recent confirmation of an association between obesity and CD has come from The Nurse’s Health Study (NHS) [40]. This is one of the largest and longest running prospective cohort studies. Starting in 1976 recruiting married nurses aged 30-55 (NHS 1) and was then expanding in 1989 recruiting women aged 25 to 42 years (NHS 2), a cohort of 238,000 nurse participants in the United States [39]. A cofounding factor however is that the recruitment was restricted to white female nurses who are likely to be highly motivated and health conscious thus not demonstrating the same degree of risk as a general population.

In the nurse cohort study among 115,374 United States women (median age 35 years), 144 cases of CD were identified. It was found that increased adiposity as measured by high BMI, waist-hip ratio and body shapes has increased incidence of CD [34]. The authors collected baseline information on anthropometrics, including: height, weight, waist and hip circumference, weight at age 18, and body shape at age 20 (using a 9 level pictogram from lean to most overweight). Using a cox proportional hazard model, hazard ratios for CD were calculated adjusting for age, smoking, physical activity, history of appendicectomy, latitude of residence, use of oral contraceptive pill, postmenopausal status and use of Non-Steroidal Anti-inflammatory Drugs (NSAIDS). The median time from enrolment to diagnosis was 9.7 years for CD. It was found that the highest BMI quartile (BMI > 30) had increased risk of CD multivariate adjusted HR 1.77 (95 % CI 1.16-2.70). Additionally, it was found that there was an association between body shape at age 20 and risk of CD, with a MV-adjusted HR of CD of 1.77 (95 % CI, 1.15-2.71) for women with overweight and obese body shape, compared to women with a thin or slender body shape [37, 40].

However, a recent cohort study of 300,724 participants found with a much smaller number of incident cases, that obesity/high BMI was not associated with the risk of development of CD [38]. Participants were men and women aged 20 to 80 years from 10 European countries without CD or UC. Recruitment took place from 1991 to 1998 and participants were followed up until 2004. Median age of CD diagnosis was 56.4 (range 24 to 78.7 years), much older than the previous two confirmatory studies with median time after recruitment of 5.1 years (range 1.5 years to 14 years) However the incident cases of CD in this study was 75 which is fewer than NHS 2 which had a total of 144 incident cases [40]. In addition the length of follow up is also comparatively shorter. It may be possible therefore that a true risk was not detected due to small number of the incident cases, and the different age range of cases meant that a different spectrum of Montreal sub-types was being studied.

### Diet

A recent systematic review of dietary factors on risk of developing and risk of relapse in IBD concluded that no firm conclusions could be drawn [45]. The authors identified 24 case-control studies which examined the intake of specific food components and CD onset. Nineteen studies reported the consumption of fruit and vegetables of which 5 studies found a significant protective effect of high overall fruit intake for the onset of CD. Six further studies found a protective effect of pre-illness high vegetable intake. Furthermore, high intake of grain-derived products was consistently shown to be negatively associated with development of CD in five studies; the authors suggested that this implied the protective role of a high fibre intake. Several studies found increased risk of CD development from high intake of carbohydrates (sugar, sugar containing foods, monosaccharides, disaccharides and starch). However the NHS I and II, did not find any association with long-term intake of total fat, saturated fats or unsaturated fats with risk of CD [16, 34]. As previously mentioned, from our study on the determinants of FC, from diet, only fibre intake would be predicted to be a risk factor for CD [20].

Shedding light on this matter is a recent report of findings from the American Nurses study, which has demonstrated that reduced fibre intake is indeed a risk factor for late onset CD [35]. This is supported by our own findings about the association of fibre intake with FC. Using data of 170,776 women, followed up over 26 years with a median age of CD diagnosis of 52 years, the authors found that the highest quintile of fibre intake (median 6.4 g/day) was associated with a lower incidence of CD. The high fibre group were more likely never to have smoked, less likely to be obese and less likely to use aspirin. This supports data from previous case-control studies that have found low consumption of fibre and high consumption of sugar to be significantly associated with the development of CD [46]. No association was found between fibre and risk of ulcerative colitis. Other components of diet that have been implicated in the past were not shown to be associated with the development of CD [47]. In the same cohort no association was found between consumption of fat and specific fatty acids [36]. This mirrors our findings on the effect of different dietary components on FC.

This recently reported study adds further support to the concept that FC is a useful surrogate marker for risk of CD development. In particular it opens the door to more detailed dietary investigation into the aetiology of CD.

### Physical inactivity

Like diet, physical activity as a risk factor for CD development in case control studies is highly prone to bias. In an early retrospective study, it was found that occupations involving outdoor work and physical activity (including brick laying and construction workers) conferred a protection against onset of IBD, whilst people in occupations associated with lower physical activity such a desk work were at greater risk of developing IBD [48]. A larger Danish retrospective cohort study looked at occupation and risk of hospitalisation with IBD. Comprising of 2 cohorts of over 2 million people it was found that people with sedentary work had a standardized hospitalisation ratio of 125 (95 % CI 116.9 – 133.1) [49]. Sedentary work was interpreted as lack of physical activity.

A small case-control study (*n* = 305) found that regular physical activity 5 years before disease onset was associated with a decreased risk ratio. Daily exercise in men conferred a statistically significant RR 0.4 (0.2-0.9). Daily exercise in women was found to have a lower risk of CD, though this was not significant [50]. This was further supported by a study that found in pre-illness IBD patients (*n* = 88) reports of physical activity was lower compared to clinical controls (*n* = 68) [51]. These studies however experience biases affecting most case-control studies.

The data from the above studies are strengthened by recent data from the American NHS which demonstrated the protective effects of physical activity [39]. The authors divided physical activity level into quintiles and found that those in highest physical activity group were at a reduced risk of developing CD compared to the lowest physical activity quintile HR 0.64 (0.44 to 0.94), after adjusting for confounders such as: body mass index, smoking, appendicectomy, oral contraceptives, NSAIDs, and postmenopausal hormone therapy.

### Smoking

Smoking is one of the first established risk factors for the development of CD [52]. The association has been confirmed repeatedly in numerous studies and has been evaluated by systematic review and meta-analysis showing smoking to confer a significant higher risk of development of CD [53]. We identified a weak but non-statistically significant trend between smoking and FC levels [19].

### Non-steroidal anti-inflammatory drugs

Regular use of NSAIDs has been associated with the risk of developing CD. The EPIC cohort found that regular aspirin use increased the risk of developing CD by 6 fold (OR = 6.14, 95 % CI = 1.76–21.35) [38].

There is also further prospective cohort data from the NHS which showed that high frequency and long duration of NSAID use, though not including aspirin, was associated with higher risk of CD occurrence [34].

### Proton pump inhibitors

The role of PPIs on the risk and effect on disease course of CD remains controversial. To date there is no prospective data on CD risk. Juillerat and colleagues found the PPI group to have increased relative risk of experiencing a mild flare compared to the non-PPI group; adjusted RR 1.18 (1.02 -1.37) [54]. More recently, it has been demonstrated in a retrospective case-control study that CD patients using PPIs were at significantly increased risk of developing severe flare-up compared to the CD PPI naïve group (OR 6.8 95 % CI 1.1 – 42.85) and also at significant risk of developing mild flare-up (OR 14 95 % CI 3.377-63.62) [55].

## Conclusion

The specificity of FC for detecting IBD could be improved by considering factors that affect levels in normal subjects. Specifically, some form of adjustment for age needs to be considered together with a history of proton pump inhibitor use.

We believe that FC is likely to prove a useful surrogate marker for risk of developing CD. It will help in exploring environmental determinants of CD, particularly diet. This review has also given a theoretical basis for considering the epidemiological determinants of CD which to date has been missing. We believe that an important part of validation of novel environmental risk factors for CD perhaps established in case control studies, should be that they have the appropriate effect on FC. It is often difficult, impossible or expensive to obtain long-term prospective data, particularly in young subjects. We believe that this concept has been validated by our identification of the association of BMI with FC, subsequent case-control confirmation and more recent confirmation in prospective cohort studies.

Further large-scale studies are called for to better characterize the determinants of FC. This may not only be important for CD but also possibly for understanding a variety of cardio-metabolic disorders as the effects of the gut microbiome on FC, metabolic and systemic inflammatory activity become better understood.

The utility of a surrogate marker for development of CD risk would be of enormous benefit. This is illustrated by the contradictory results of studies of diet and other environmental agents into the pathogenesis of disease onset. Previously gut permeability has been considered as a possible surrogate marker, but the difficulty in its measurement has precluded large-scale studies.

A small number of recent prospective studies support the potential utility of FC as a surrogate marker of development of CD risk. FC has also proved its utility in the discovery of a novel and unexpected risk factor for CD; namely, obesity.

Large-scale population based studies are called for to investigate the determinants of FC in otherwise healthy subjects for a number of reasons. Not least, a better understanding of the determinants of normal levels will improve the specificity of the test. This is important, as the end result of a positive FC, is a colonoscopy; an invasive and expensive test. Secondly, there are no other better candidates as a surrogate marker for risk of CD development and it will provide a better understanding of the pathogenesis of CD. If the findings from our original population based study are replicated, then Abbreviationsfuture candidate risk factors can be assessed for their plausibility not only through case-control studies, with their inherent unreliability but also through their effects on FC.

## Abbreviations

BMI: Body mass index
CD: Crohn’s disease
FC: Faecal calprotectin
IBD: Inflammatory bowel disease
NHS: Nurses health study
PPI: Proton pump inhibitor
UC: Ulcerative colitis
